# Unsupervised novelty detection for time series using a deep learning approach

**DOI:** 10.1016/j.heliyon.2024.e25394

**Published:** 2024-02-01

**Authors:** Md Jakir Hossen, Jesmeen Mohd Zebaral Hoque, Nor Azlina binti Abdul Aziz, Thirumalaimuthu Thirumalaiappan Ramanathan, Joseph Emerson Raja

**Affiliations:** a Faculty of Engineering and Technology, Multimedia University, Melaka, Malaysia; b Faculty of Information Science and Technology, Multimedia University, Melaka, Malaysia

**Keywords:** Anomaly detection, SHS, LSTM, DCNN

## Abstract

In the Smart Homes and IoT devices era, abundant available data offers immense potential for enhancing system intelligence. However, the need for effective anomaly detection models to identify and rectify unusual data and behaviors within Smart Home Systems (SHS) remains a critical challenge. This research delves into the relatively unexplored domain of novelty anomaly detection, particularly in the context of unlabeled datasets. Introducing the novel DeepMaly method, this approach provides a practical tool for SHS developers. Functioning seamlessly in an unsupervised manner, DeepMaly distinguishes between seasonal and actual anomalies through a unique process of training on unlabeled pristine features extracted from time series data. Leveraging a combination of Long Short-Term Memory (LSTM) and Deep Convolutional Neural Network (DCNN), the model is primed to detect anomalies in real-time. The research culminates in a comprehensive data prediction and classification process into normal and abnormal data based on specified anomaly thresholds and fraction percentages. Notably, this function operates seamlessly unsupervised, eliminating the need for labeled datasets. The study concludes with a complete data forecasting and sorting method that divides data into normal and abnormal categories based on defined anomaly thresholds and fraction percentages. Working in an unsupervised mode reduces the requirement for labeled datasets. The results highlight the model's prowess in new detection, which has been successfully applied to benchmark datasets. However, there is a restriction since deep learning algorithms can recognize noise as abnormalities. Finally, the investigation enhances SHS anomaly detection, providing a crucial tool for real-time anomaly identification in the ever-changing IoT and Smart Homes scene.

## 1 Introduction

Due to its pervasiveness, anomaly detection has long been among the most essential crucial study fields. We notice the irregularities that are the center of our attention in everyday life. IoT, or the Internet of Things has a great significant impact on civilizing the whole world The number of devices associated with IoT has already reached around US$16.7 billion in 2018, a 94 % growth rate from 2017 and is expected to increase rapidly in the coming years to meet up the global demand[1]. Various smart city applications link massive IoT devices to real-world items, to significantly benefit city life [2]. These large numbers of IoT schemes provide distinct services and technologies that will produce probable complications in the IoT networking management system [3].

The phenomenon that significantly diverts from the normal conditions is considered an anomaly. Meanwhile, the approach that identifies the data points apart from the normal distribution in the dataset is known as anomaly detection. It is critical to discover abnormalities as soon as possible to avert major concerns such as a financial system hack and total equipment failure. Companies from various logistics and manufacturing industries are devoting significant resources to gathering large amounts of data and analyzing anomalous patterns to serve their consumers better. These data are the time series for most of the cases. It is very difficult and challenging to identify the abnormalities from these data types as they contain inherent properties of irregularities, periodicity, and seasonality. It is also essential to identify the seasonal anomaly and actual anomaly.

In this work, we utilize an unsupervised technique to overcome the challenge of classifying vast amounts of data in real-life settings. Though distinct unsupervised approaches were used to recognize anomalies, the inherent properties' time series remains unfocused. Again, conventional anomaly detection techniques such as distance-based approaches do not address the proper contextual properties of time series data. Hence, it has become more challenging to find out the point abnormalities.

Distinct methods were used to detect the anomalies, especially in the time-series dataset. The most common technique for detecting anomalies is statistical anomaly detection. The KNN anomaly identification approach is the most extensively used and simplest form of unsupervised anomaly detection technique [4,5]. This approach uses the k-nearest-neighbours distance to generate the anomaly score. However, this approach vastly depends on K. The failure may occur during typical point data, which has a scarcity of neighboring points. Selecting the correct threshold value for KNN can be a non-trivial task as it necessitates a deep understanding of the signal's properties.

The local Outlier Factor technique based on the local density is another popular unsupervised algorithm for anomaly identification[6,7]. The neighboring data point is intended to distribute crucial spherically, which is this technique's key premise. Estimating spherical density has become inappropriate when the distributed data are placed linearly.

Histogram Based Outlier Score or HBOS is also considered an unsupervised algorithm for anomaly detection based on statistical characteristics [8]. Compared to the clustering and neighboring-focused approaches for anomaly detection, this algorithm is significantly less computationally expensive. HBOS can perform both cases by providing dynamic and constant bin-width histograms [9]. This technique does not require any prior training data and tries to determine a decision boundary that separating the points and the origin as much as possible. This method is also used to discover irregular daily behaviors such as sleeping, sitting, and walking habits [10]. Laptev et al. [11] introduced a scalable and generic method to detect anomalies in the time-series large-scale data. They have utilized a collection of anomalies forecasting approaches, including an anomaly filter process to detect anomalies in time-series data. They have obtained around 60 % enhancement in recall and precision.

Few research has integrated [[12], [13], [14]] OCSVM-focused anomaly detection algorithm which is known as One-Class Support Vector Machine for detecting abnormalities. There are 6 Meta features introduced through this approach on the actual multivariate or unilabiate time series data. After that, anomalous states were discovered by applying the OCSVM to the data space based on the Meta feature. An outlier detection method named Support Vector Data Description (SVDD) was introduced by Liu et al. (Liu et al., 2012). OC-SVM may not perform well on datasets with complex, non-linear boundaries. Moreover, OC-SVM can be computationally expensive, primarily mainly when applied to large datasets.

The principal Component Analysis (PCA) dependent method was suggested by Shyu et al. [15] to detect the anomalies where minor and significant principal components from the normal instances were utilized in the predictive model. In order to calculate the covariance and correlation matrices, the Minimum Covariance Determinant technique was applied instead of the conventional approach, which was a close variant of the previous technique [16]. The statistical-based Autoregressive Moving Average and its modification have been used to forecast the time series data and anomaly detection. For instance, Yu et al. [17] utilized an autoregressive moving average-focused anomaly detection system in wireless sensing networks for traffic control purposes. They claimed that using a short-step exponential weighted average approach to judge network traffic anomalies is the key to making better decisions. Yaacob et al. [18] suggested a method for detecting Denial-of-Service (DoS) assaults early on. Anomalies in network traffic are found by comparing real network traffic to ARIMA's expected patterns. For time series analysis, various ANN architectures have been successfully used. Malhotra et al. [19] suggested an anomaly detection strategy based on layered LSTMs. The prediction algorithm was trained on the normal time stamps, and the error vectors for specified sequences were computed using the model. The particular time series sequences are classified into anomalous and normal or based on the error threshold. Chauhan et al. [20]adopted a similar approach to detect irregularities in ECG data. They employed a combination of RNN and LSTM to detect four different anomalies. Kanarachos et al. [21] introduced a deep-learning anomaly detection system that combines Hilbert transforms and wavelets with neural networks. It was supposed to find out whether there are any anomalies in time series trends. LSTM was used by Lipton et al. [22] to categorize a time series as aberrant or normal. Convolution Neural Network (CNN) was employed by Zheng et al. [23] for categorising time series data with multivariate properties. Multi-channel DNNN was proposed to take a single-dimensional from multivariate data as input and train on separate features for an individual channel. After that, a single layer of multi-layer perceptron (MLP) was utilized to compute the classification. Schreyer et al. [24] employed a deep auto-encoder to find out the anomalies from the long-range accountable dataset.

Numerous studies have achieved important advancements in the field of time-series anomaly detection, focusing on either novelty identification or outlier detection. Researchers have utilized several methods, including deep learning techniques like autoencoders [25], for novelty detection-focused investigations. They are leveraging the advantages of unsupervised learning and featureless data to uncover unique temporal patterns. The problem of finding abnormalities in featureless data has drawn several researchers' attention [26,27] which, although resource-effective, might not be able to detect intricate patterns in time-series data. These novelty detection techniques frequently offer insightful information on comprehending temporal behaviours that are just beginning to occur.

On the other hand, outlier detection-focused research has also advanced significantly [28]. developed a strategy based on deep learning and autoencoders, attaining a high accuracy rate of 96.77 % in spotting outliers, and [29] built a model with a 97.66 % accuracy rate for outlier identification. For purposes like fraud detection and quality control, such systems strongly emphasise spotting deviations from the norm. However, some of these studies do not include thorough comparative analyses, making it difficult to evaluate how well they perform compared to other approaches [30,31]. In outcome, these research projects add to the expanding tools of time-series anomaly detection techniques by meeting the complex needs of novelty identification and the realistic expectations of outlier detection.

Currently, a variety of currently used anomaly detection approaches are being investigated, with a focus on time-series datasets, which are common in SHS. These techniques included statistical anomaly detection using KNN, LOF, and HBOS algorithms. The study also explored PCA-based techniques and machine learning-based techniques including OCSVM and SVDD. Recurrent neural networks (RNNs), LSTM, and CNNs were also studied, as well as autoregressive moving average models. However, the relevance of these existing methods lies in their applicability to anomaly detection, a critical challenge in SHS where unusual data or behaviors need to be identified and addressed. However, these approaches have limitations in handling univariant time-series datasets and providing a robust distinction between seasonal and actual anomalies.

The research-proposed approach overcomes these difficulties by including a feature SHS programmers may use easily. This function considers asymmetrical time-series data and distinguishes between various anomalies, such as seasonal changes and actual anomalies. It uses an LSTM and a DCNN, which are helpful in capturing temporal relationships and hierarchical characteristics in time-series data and are thus well suited for the SHS context. This function's capacity to operate unsupervised eliminates the requirement for labeled data, which has practical advantages. By giving developers a flexible tool for anomaly detection in SHS, this study closes a critical gap in currently available solutions. It advances the development of more intelligent, more effective home automation systems.

This research introduces a novel approach to anomaly detection in time series data, emphasizing the use of unsupervised deep learning techniques. While traditional anomaly detection methods often rely on labeled anomaly data, our approach innovatively shifts the focus to unsupervised learning, allowing for the detection of anomalies without the need for prior labeled information. The primary contributions of this work include developing and evaluating deep learning-based models, such as CNN and LSTM, for unsupervised anomaly detection. Through comprehensive experiments and evaluations on diverse datasets, the effectiveness and adaptability of our proposed methods were demonstrated. The significance of this work lies in its potential to advance the field of anomaly detection by providing accurate, flexible, and data-driven solutions for real-world applications where labeled anomaly data is limited or unavailable.

## 2 Methodology

In many domains, such as object Web threat detection [32], IoT intrusion prevention systems [33], IoT network activity monitoring [34], aerial monitoring systems [35], and more, the use of a DNN in anomaly detection has dramatically increased. The anomaly detection technique, depending on deep learning for time-series data, is provided in this article by extending the unsupervised novel, an integrated unsupervised learning system trained exclusively on the standard dataset. The input from the deep learning algorithm is independent of the last sequential values.

**Fig. 1:**
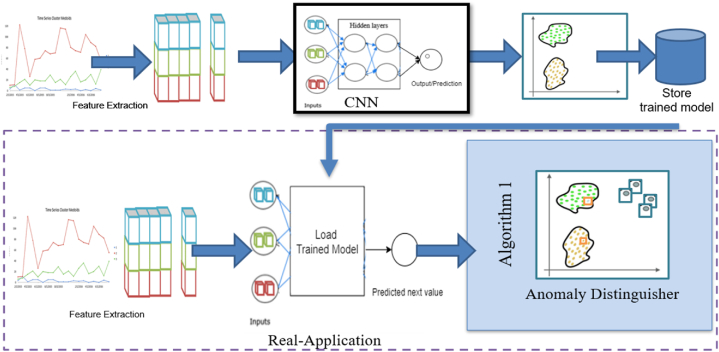
The proposed approach for unsupervised novelty detection using deep learning.

Furthermore, the algorithm was trained on a subset that tends to be expected rather than training on the entire dataset. As a result, this approach cannot be used directly in the analysis of the time-series dataset since the chronological sequence dataset with no gaps in time is required in that case. As indicated in Fig. 1, the suggested solution in this work was meant to address these issues and comprises two perspective segments of algorithms: a time-series predicting and an anomaly distinguisher algorithm.

The retrieved characteristics were used to train a forecasting model, which was used to anticipate the following predictions. MLP, LSTM, and CNN models were compared as predicting models, and the best one was chosen. These approaches benefit from being high-performing time series models that may be applied directly to time series without the need for feature engineering. However, to allow seasonal identification, time-domain data was taken first. Furthermore, because the forecasting model's precision directly impacts the effectiveness of novelty anomaly detection, it was ensured that the model was maintained with the greatest accuracy and the least amount of error. Even though there was no usage of the labeled dataset in this example, the system was assessed by the accuracy and loss of the learning model in a supervised way. In other words, because the idea of novelty detection necessitates the discovery of new unknown anomalies when the whole subsequences are utilized to learn the deep model, the model is better suited for normal data rather than for abnormal data.

Rather than utilizing the fundamental clustering technique, stages of the approach were given in the anomaly distinguisher module. According to the module, if the anticipated value is outside of the confidence interval, the sample is labeled as an anomaly. On the other hand, estimating the confidence interval is not a simple task. The estimation is based on the anomaly % parameter, which is the number of anomalies predicted to be seen.

### 2.1 Effective deep time-series forecasting model

A deep learning-based abnormalities detection technique named DeepMaly is a predicting algorithm that is based on CNN. CNN is a branch of Artificial Intelligence that can be applied in distinct types of applications for its parametric efficiency, particularly in NLP (Natural Language Processing) and CV (Computer Vision). As it processes a mathematical model named convolution, it is rightly named after that particular model. The CNN network consists of many layers such as convolution and pooling, and all the layers are connected to each other in the network. Typically, each convolutional layer contains two phases. The layer executes the convolution operation in the first step and gives the result of its linear activations. Afterwards, every linear activation must undergo a non-linear activation. Convolution is a mathematical process that produces a third function by combining two functions with real-valued parameters.

Time series datasets have unique characteristics; hence it was chosen to employ CNNs for unsupervised novelty discovery in these datasets. Because of its intrinsic simplicity, conventional CNNs are a better fit for these datasets since they place more of a focus on temporal dependencies than spatial patterns. When used for time series data rather than high-resolution image data, improved CNN models usually only result in marginal performance gains for novelty recognition. For datasets with limited resources, traditional CNNs are advantageous because they are computationally efficient and more flexible to various input dimensions. The most fundamental benefit of traditional CNNs is that they provide a clear and intelligible framework for feature extraction, which is essential for describing model selections in industries like finance or healthcare.

**Fig. 2:**
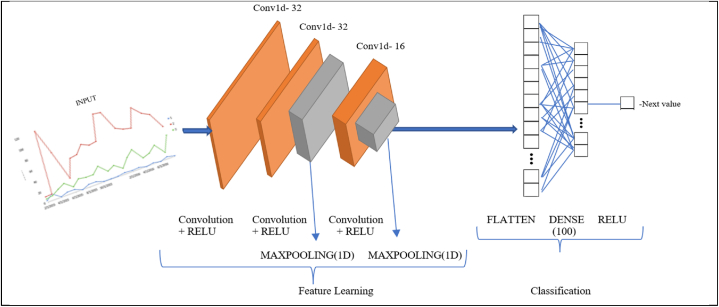
Convolution network architecture for novelty detection time series data.

To complete the architecture and its hyperparameters, we conducted extensive tests. The architecture of the proposed network consists of two convolutional layers with a max-pooling layer of each as shown in Fig. 2. The data was tuned into vectors of w which gave a clear indication of having the same number of inputs in the input nodes of the proposed network. The first two convolution layers consist of 32 filters. After that, an element-wise rectified linear unit (ReLU) activation function was utilized which presented in equation (1). The next convolutional layer is made up of 16 filters that use ReLU activation. Finally, all the preceding layers are connected to each neuron through a Fully Connected (FC) layer, the last layer of the network. The last FC layer is responsible for forecasting the upcoming timestamp.

The output layer has the same number of nodes as p w. In our scenario, the output node will be 1 because the forecast in timestamp is 1.

(1) Function,f(x)=max(0,x)

The disparity between the anticipated ${\hat{y}}_{j}$ output to evaluate the suggested CNN design and real $y_{j}$ was verified by the MAE (Mean Absolute Error) which is mentioned in equation (2). The behavior of the time-series data can be anticipated by lowering the error between the actual and projected values. Based on the training data, we normalized each time series.

(2) $$\text{MAE}=\frac{1}{n}\sum_{j=1}^{n}\;\left|y_{j}-{\hat{y}}_{j}\right|$$

### 2.2 Anomaly distinguisher

An algorithm was implemented for clustering the anomalies with threshold-based. This threshold value is calculated automatically using the dataset's user-defined anomaly percentage.

**Table:** 

Algorithm1
Step 1: Initialize an empty list ‘diff’ to store absolute differences. Calculate the length of ‘diff’ and store it in ‘DiffLength'. Step 2: # Selection of the most distant values AnomalyPercentage = user-defined percentage AnomalyCount = int(AnomalyPercentage * DiffLength) # Calculate threshold for anomaly detection Sort ‘diff’ in ascending order threshold = diff[DiffLength - AnomalyCount] # Find anomalies Initialize an empty list ‘AnomalyOutput' For each value in ‘diff': If value ≥ threshold: Append 1 to ‘AnomalyOutput’ # Anomaly detected Else: Append 0 to ‘AnomalyOutput’ # Normal data Return ‘AnomalyOutput’ as the result.

### 2.3 Dataset description

The dataset that is used in this research for quantitative evaluation is described in this section. Two labeled publicly available time-series datasets were shortlisted for this research. It can be noted that only anomalies containing the dataset are considered in this study.

A Numenta Anomaly Benchmark (NAB) [36].

**Fig. 3:**
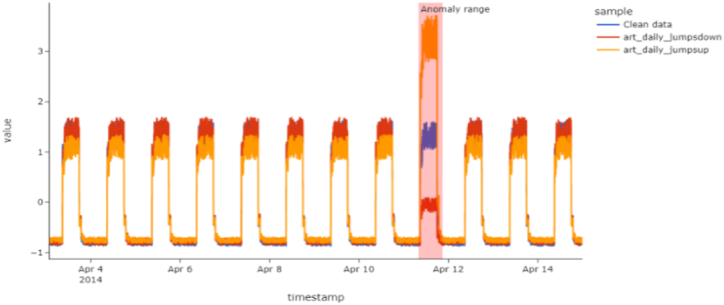
Sample time series from each set in a time-series graph.

The unsupervised learning was taught using a benchmark dataset known as NAB [36]. The NAB dataset was used in this case. (a)The three datasets utilized are art (a)daily jump down, (b)art daily jump up, and (c) art daily jump up with no anomalies. These three datasets are time-series data, except for art daily data, which contains data sets with and without anomalies. This set of data will aid in confirming the capability of detecting unique anomalies in the system. The NAB dataset is open to the public and Fig. 3 shows the time series examples from the three sub-benchmarks. Orange, blue, and red marks show the streaming of actual data whereas red marks indicate the point of abnormalities. It is very rare to find out the point abnormalities labels as it is not publicly available as a streaming dataset, which was the primary rationale for choosing this data set for the examination. Another season differs in that it has data with and without abnormalities. It was necessary to train the model using clean data before detecting the unknown anomalies in the instance of novelty detection.

B Yahoo Webscope [37].

**Fig. 4:**
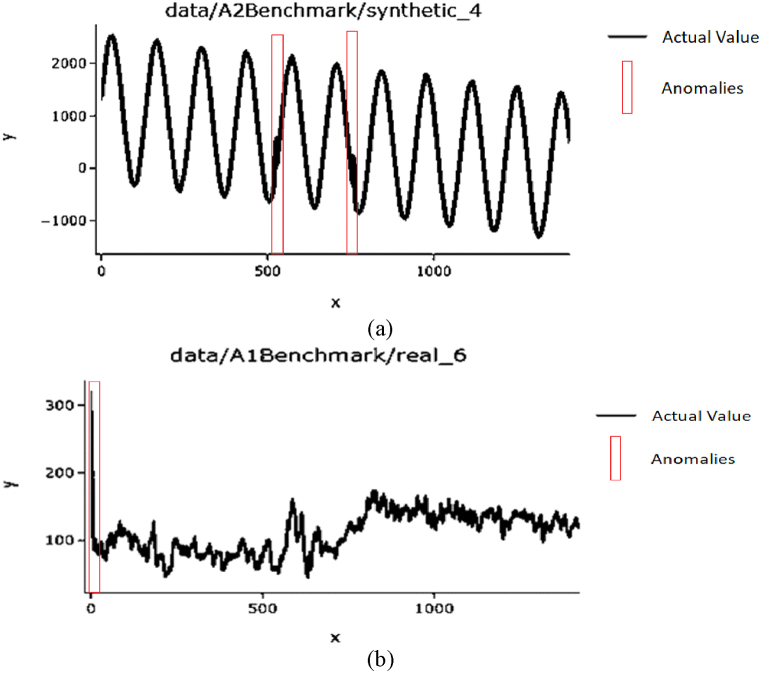
Examples of time-series from the Yahoo Webscope (a) synthetic data and (b) real data.

Yahoo Webscope is a freely accessible streaming data collection that includes real and synthetic data. The login data of Yahoo membership is included in the cleaned real data. Then, the dataset is categorized into four categories from A1 to A4. Fig. 4(a) and (b) represent examples of this time-series dataset. The timestamps of the dataset are monitored by hours and marked the anomalies based on the guideline given by the Yahoo S5 dataset. The anomalies’ range was represented by the red rectangle in Fig. 4. Outliers are contained in the A1 to A3 categories whereas change-point anomalies were contained in the A4 category. This data collection has 367-time series, each of which contains 1420 1680 occurrences. The A1 and A2 categories contain synthesis and real data respectively which are the outcome of some part of the Yahoo! Properties. These two benchmark categories are applied to validate the proposed anomaly detection approach.

## 3 Result and discussion

In this research, the concept of novelty detection was focused on. There is a difference between the process of novelty detection and anomaly detection. The training dataset consists of normal observations in novelty detection and decides whether the newly received observation fits the data. In this case, the DeepMaly model is an excellent option for novelty detection problems.

**Fig. 5:**
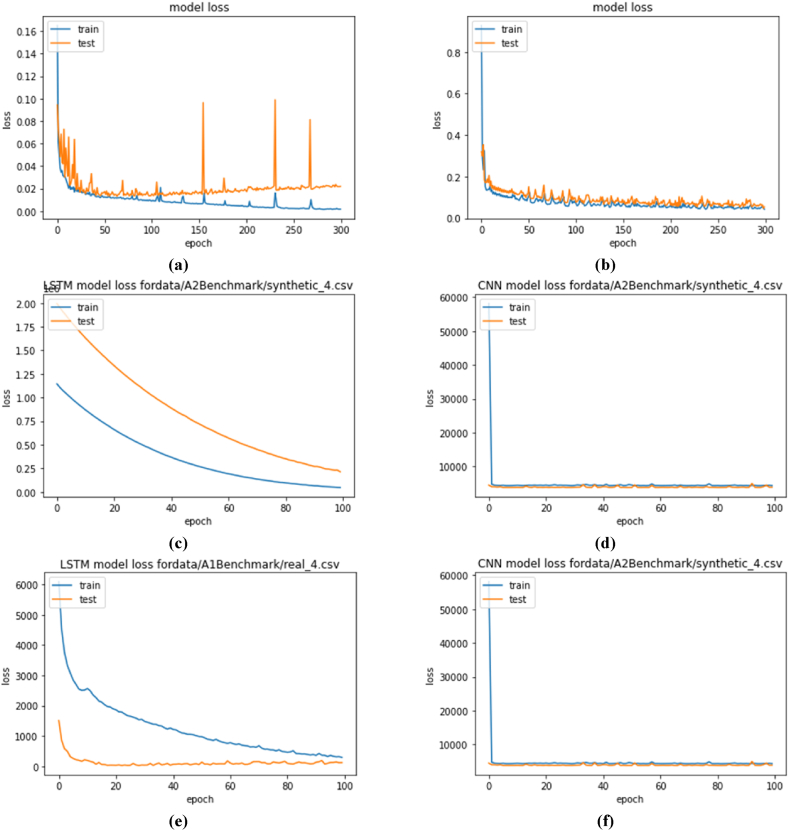

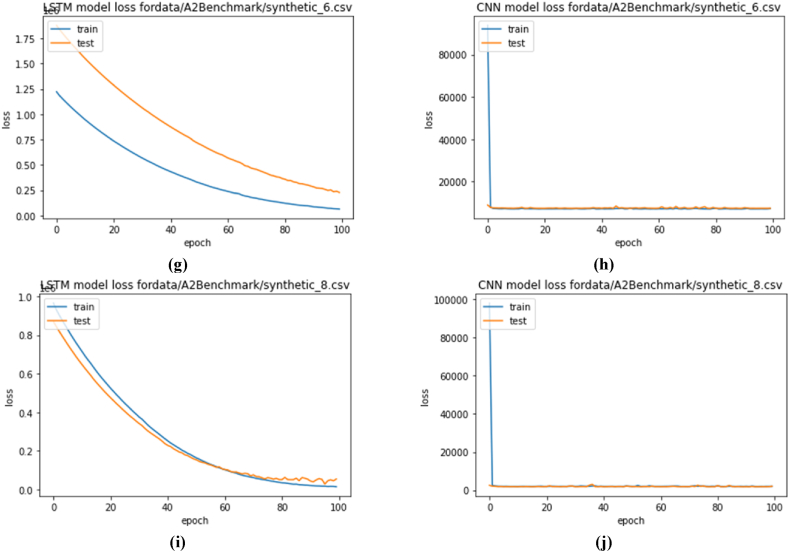
Model loss while training deep learning models: (a)using LSTM for NAB data, (b) using CNN for NAB data, (c) using LSTM for yahoo synthetic 4 data, (d) using CNN for yahoo synthetic 4 data, (e) using LSTM for yahoo real 4 data, (f) using CNN for yahoo real 4 data, (g), using LSTM for yahoo synthetic 6 data (h) using CNN for yahoo synthetic 4 data, (i) using LSTM for yahoo synthetic 8 data, (j) using CNN for yahoo synthetic 8 data.

As there are two modules, the effectiveness of the modules was tested differently. Compilation time and model loss were generated to find the effective neural network learning model for time series. Fig. 5 plots the output of model loss for the two deep learning models, and Table 1 tabulated training time.

**Table 1:** Compilation time while training deep learning models.

Neural Network	Model Loss	Training time (seconds)
LSTM	NAB dataset	607.32395
CNN		121.43911
LSTM	A2Benchmark dataset syntethic_4	228.9773
CNN		20.7954
LSTM	A1Benchmark dataset real_4	228.2449
CNN		26.3781
LSTM	A2Benchmark dataset syntethic_6	221.654
CNN		10.3206
LSTM	A2Benchmark dataset syntethic_8	221.4504
CNN		12.6697

The plot presented in Fig. 5 (a) and Fig. 5 (b) is for the NAB dataset using LSTM and CNN respectively; it is training the model without anomalies. It can be seen that LSTM is not a smooth curve, where it contains a sudden error in some epochs. Whereas CNN tends to provide the model with the least error. It has important implications for understanding the behavior and performance of these models in the context of time series data.

The LSTM model often has trouble detecting complex patterns in the time series data when there are sharp variances in the training process. These complex patterns could include abrupt shifts or abnormalities in the data, including sharp spikes or unexpected moves from the predicted trend. Additionally, the existence of unexpected mistakes may indicate difficulties in understanding and upholding long-term relationships within the sequential data. When LSTM models struggle to capture these long-range dependencies successfully, it can influence how well they perform. Furthermore, unexpected errors could be a sign of overfitting, where the model begins to match data noise, or vanishing gradient problems, where gradients are too small during training and obstruct learning. These findings highlight the difficulty of modeling time series data and the need for careful model selection, hyperparameter tuning, and regularization techniques to overcome these difficulties and improve the model's ability to detect underlying dependencies and patterns in the data.

In capturing local patterns and characteristics in the data, CNNs show valuable adaptability. Because of this feature, they are especially well-suited to situations where the time series data displays softer trends or dependable local patterns. In these situations, CNNs can produce models with more stability and reduced error rates, improving their applicability for jobs like time series forecasting. CNNs also exhibit a remarkable resistance to noise and slight variations in the data. They are sufficiently robust to endure the presence of noisy data or slight abnormalities. This robustness is especially helpful in practical situations where time series data frequently contains noise from numerous sources. CNNs are an excellent choice for applications where data consistency and the capacity to tolerate noise are crucial factors since they can produce smoother curves even in the presence of noise. This helps with their dependability and predicted accuracy.

Sudden errors in LSTM training highlight the challenges in capturing complex temporal dependencies. At the same time, the smoother curves in CNN suggest a better ability to handle local patterns and noise. Understanding these implications can guide the selection of the appropriate model architecture and inform strategies for model improvement, regularization, and data preprocessing to enhance performance in time series forecasting and anomaly detection tasks.

From Tables 1 and it can be seen that CNN takes less training time than LSTM, which means CNN trains faster. There are numerous fundamental reasons why LSTM models frequently require longer training times. First of all, LSTM models are more sophisticated than other models. LSTMs integrate complex internal processes intended to capture sequential relationships in data as recurrent neural networks (RNNs). These techniques entail learning and updating internal states across sequences, which might be intrinsically more difficult and time-consuming than the operations of neural network topologies with simpler neural networks, such as feedforward networks. Second, the lengthened training time of LSTM models is significantly influenced by gradient flow. Deep learning models are vulnerable to gradient vanishing or ballooning problems, especially recurrent ones like LSTMs. Gradients can become abnormally tiny (vanishing) or excessively big (exploding) during the backpropagation process, hindering the model's convergence and requiring extra training epochs to stabilise the learning process. To ensure practical training, addressing these gradient-related issues requires careful initialization methods, gradient clipping, and architectural adjustments.

In Table 1, the percentages representing training time disparities are shown as follows:

• Compared to the LSTM model, the CNN model used around 19.96 % less time on the NAB dataset.
• The CNN model trained on the A2Benchmark dataset synthetic_4 in around 9.08 % of the time needed by the LSTM model.
• The CNN model's training time on the A1Benchmark dataset real_4 was around 11.57 % slower than the LSTM models.
• The CNN model trained on the A2Benchmark dataset synthetic_6 in around 4.66 % of the time it took the LSTM model.
• The CNN model's training time on the A2Benchmark dataset synthetic_8 was around 5.71 % slower than the LSTM models.

These percentages demonstrate the stark disparity in training durations, with CNN consistently outperforming LSTM across various datasets.

For further evaluation, another data from the Yahoo dataset was obtained; the loss output is presented in Fig. 5 (c)-Fig. 5 (j). The training output using LSTM is Fig. 5(c)–. 5 (e), Fig. 5 (g), and Fig. 5 (i); the training output using CNN is presented in Fig. 5(d)–. 5 (f), Fig. 5 (h) and Fig. 5 (j). LSTM required more than 100 epochs to get a minimum error. However, considering 100 epochs for deep learning, LSTM takes a longer training time than CNN for every dataset. The number of epochs in training a deep learning model holds a pivotal role in determining its effectiveness, but it also brings about certain critical considerations. One of the foremost concerns is the risk of overfitting. When the number of epochs is increased without vigilant monitoring of the model's performance, it raises the potential for overfitting. Overfitting is a scenario where the model becomes excessively specialized in memorizing the intricacies of the training data, rather than genuinely learning, and generalizing from it. The consequence of overfitting is a model that performs exceptionally well on the training data but poorly on unseen or new data, undermining its real-world utility. Various regularization techniques and strategies such as early stopping are often employed to combat overfitting. These methods help strike a balance between training for an adequate number of epochs to capture essential patterns and preventing the model from becoming overly specialized.

However, it's essential to recognize that an increase in the number of epochs comes at a computational cost. Training a deep learning model for an extended period can be computationally expensive and time-consuming. The process demands substantial computational resources and time investments, including high-performance hardware like GPUs or TPUs. This computational intensity can render prolonged training impractical for certain applications or on resource-constrained devices, limiting the model's deployment ability. Therefore, practitioners often must strike a careful balance between training long enough to capture valuable insights from the data and ensuring efficient resource utilization, particularly when deploying models in real-world scenarios with constraints.

### 3.1 Anomaly distinguisher output

**Fig. 6:**
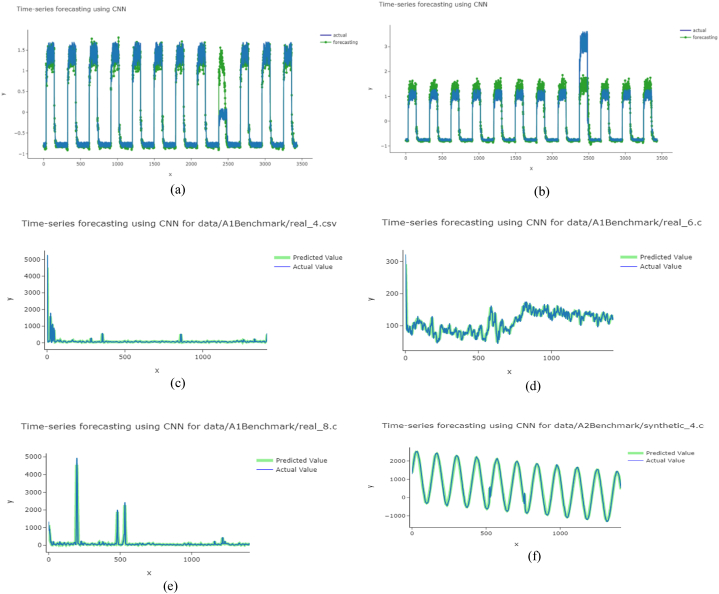
Time-series forecasting using CNN: (a) NAB down data, (b)NAB sudden jump data, (c)yahoo real 4, (d) yahoo real 6, (e) yahoo real 8, (f)yahoo synthetic 4.

Once the trained model was obtained, it was further used for forecasting. While forecasting, it predicts the following sequence of data. A sample of forecasting and the actual value is presented in Fig. 6. The blue light indicates the actual data, and the green line indicates forecasted data. This data set is passed through algorithm 1 to separate normal data from abnormal data. Fig. 6(a) and (b) present the actual and forecasted output. The actual data pattern is seasonal; it detects output according to the time frame. It can be clearly seen that data around 2400–2500 have been down and up to date in Fig. 6(a) and (b), respectively. However, the prediction detects the actual value. Later, this value difference was used to find the anomalies in the time series.

Similarly, for the time series Yahoo Webscope for real and synthetic data, some examples are presented in Fig. 6(c)–6(f). It can also be visualized that the actual and predicted values are very similar, which helps detect anomalies. But in this case, error calculation was used rather than distance.

### 3.2 Predicted output

**Fig. 7:**
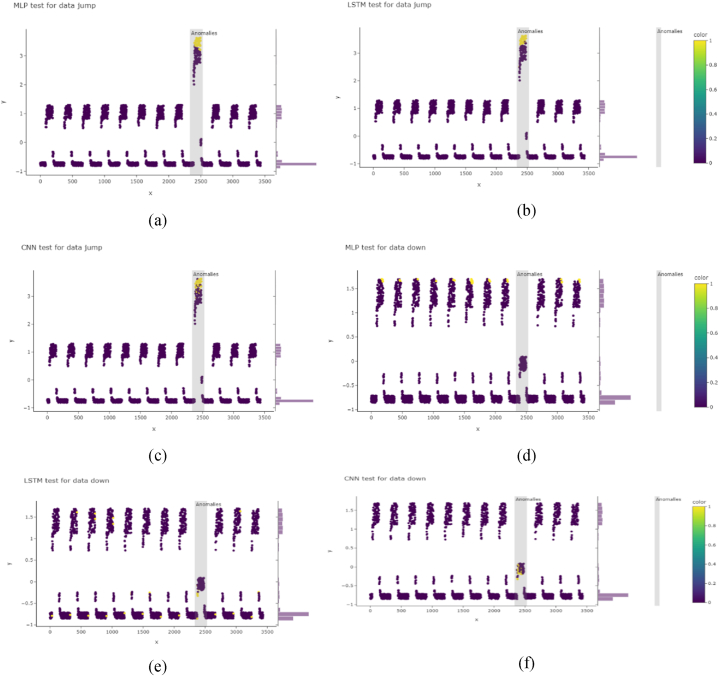
Scatter plot of detecting anomalies for NAB data (a) jump using MLP, (b) jump using LSTM, (c) jump using CNN, (d) down using MLP, (e) down using LSTM, (f) down using CNN.

The detected anomalies using the anomaly distinguisher (algorithm 1) are presented here. The values were plotted in a graph (Fig. 7), where yellow indicates anomalies, and others are considered normal data. For data jump, all three trained models (MLP, LSTM, and CNN) detected both the anomaly points in the correct location. However, it was difficult for MLP and LSTM to detect correctly for the down dataset. Here, CNN tends to work best. In the case of MLP, it can be seen that it continuously detects more excellent values as anomalies, but it is not an ideal case; a lower value can also be an anomaly.

**Fig. 8:**
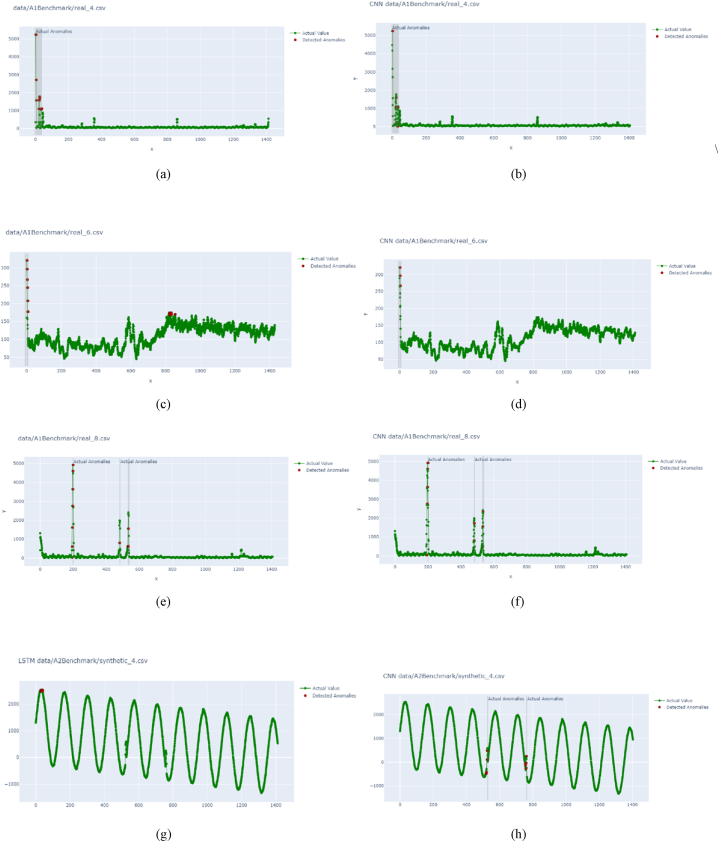

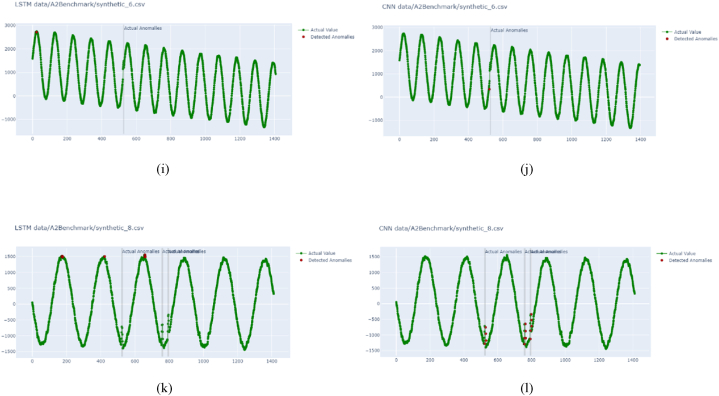
Anomaly detection on time series Yahoo Webscope for real and synthetic data: (a) using LSTM for yahoo real 4 data, (b) using CNN for yahoo real 4 data, (c) using LSTM for yahoo real 6 data, (d) using CNN for yahoo real 6 data, (e) using LSTM for yahoo real 8 data, (f) using CNN for yahoo real 8 data, (g) using LSTM for yahoo synthetic 4 data, (h) using CNN for yahoo synthetic 4 data, (i) using LSTM for yahoo synthetic 6 data, (j) using CNN for yahoo synthetic 6 data, (k) using LSTM for yahoo synthetic 8 data, (l) using CNN for yahoo synthetic 8 data

On evaluating the trained model from Fig. 8, the trained models were further evaluated using Yahoo benchmark data. Here also, CNN tends to work better than LSTM. For example, for real data, LSTM output in Fig. 8 (a and e) and CNN output in Fig. 8 (b and f) detected anomalies in the correct position, whereas LSTM detection using another set-in Fig. 8 (c) indicates two area detection, where one position is false positive detection and CNN detection in Fig. 8 (d) showing correct detections. LSTM tends to detect all false-positive anomalies for synthetic data, and CNN works better here.

The choice for anomaly detection in time series data was determined on the objectives and limits of the application since the training time difference between LSTM and CNN architectures has a major influence. To begin with, training time becomes a crucial factor in situations where real-time or almost real-time anomaly detection is important. Compared to LSTMs, CNNs often require less training time because of their simpler design and parallel processing capability. CNN may be the most appropriate choice for quick anomaly detection since it ensures prompt reactions to changes in the time series data. Second, the selected design must accommodate the computing resources that are readily accessible. It can be computationally expensive to train LSTMs, especially on big and complicated datasets, and high-performance hardware like GPUs or TPUs may be required. On the other hand, CNNs are frequently more computationally effective, making them a viable choice for applications with limited resources where training time and computational cost must be controlled.

The results of a CNN applied to a collection of 20 data subsets taken from Yahoo are shown in Table 2 as an aggregated performance. Several criteria are used to assess how well CNN detects abnormalities in time series data. The model typically exhibits a precision of 0.61, indicating that around 61 % of CNN's optimistic predictions are correct. The average recall, also referred to as sensitivity, is 0.7930, meaning that the model accurately recognised 79.30 % of the real anomalies. The average F1-Score, which balances recall and accuracy, is almost 0.5999, suggesting a successful trade-off between these two crucial factors.

**Table 2:** Aggregated performance of CNN for anomaly detection in 20 yahoo data subsets.

Data Subset	Precision	Recall (Sensitivity)	F1-Score	Accuracy
1	0.8	1	0.8888	0.9992
2	1	0.5555	0.7142	0.9971
3	0.2	1	0.3333	0.9971
4	0.8	1	0.8888	0.9992
5	1	0.5555	0.7142	0.9971
6	0.2	1	0.3333	0.9971
7	0.8	1	0.8888	0.9992
8	1	0.5555	0.7142	0.9971
9	0.2	1	0.3333	0.9971
10	0.8	1	0.8888	0.9992
11	0.2	0.1111	0.1428	0.9915
12	0.2	1	0.3333	0.9971
13	0.6	0.75	0.6666	0.9978
14	1	0.5555	0.7142	0.9971
15	0.2	1	0.3333	0.9971
16	0.8	1	0.8888	0.9992
17	1	0.5555	0.7142	0.9971
18	0.2	1	0.3333	0.9971
19	0.8	1	0.8888	0.9992
20	0.4	0.2222	0.2857	0.9929
**Average**	**0.61**	**0.7930**	**0.5999**	**0.9972**

Additionally, there is a trade-off between training time and model accuracy. LSTMs are excellent at capturing subtle temporal patterns and long-term relationships, which might improve detection accuracy. The lengthier training period of LSTMs may be acceptable if reaching the highest level of accuracy is the main objective and computing resources and real-time restrictions permit. On the other hand, if effectiveness and speed of reaction are priorities, a CNN might offer a practical compromise between accuracy and speed.

### 3.3 Compared with current Unsupervised Learning techniques for anomaly detection

In Table 3 comparison of several studies examining the field of time-series data analysis, focusing on the use of deep learning approaches for detecting skills is presented. The table's rows each represent a different research study, illuminating the salient features of each one. The references to these study projects are painstakingly listed in the “Reference” column, making it simple to access them for more investigation. Notably, the “Time-Series Data” column offers a convenient quick reference, highlighting which research focuses on time-series data analysis, a crucial step in anomaly identification in dynamic situations. An aspect related to modern anomaly detection strategies, the “Deep Learning” column, which simply highlights the usage of deep learning techniques in each research article, is of exceptional value. The “Comparative Study” column also provides information on whether these researchers use comparative analyses, which are crucial for benchmarking and comprehending the efficacy of their suggested strategies. With careful distinction between “novelty” and “outlier” detection, the “Insight” column articulates the main goal of each study project. Finally, the “Accuracy (%)" column offers a numerical measurement, where available, allowing researchers and readers to assess the methodology's level of robustness and the claimed accuracy rates.

**Table 3:** Comparison of current researchers on Time-Series Data Analysis and Detection Capabilities.

Reference	Time-Series Data	unlabeled Data	Featureless	Deep learning	Comparative study	Insight	Accuracy (%)
[26]	✓	✓	X	✓	✓	novelty	N/A
[28]	✓	✓	✓	✓AE	✓	outlier	96.77
[31]	✓	✓	✓	✓AE	X	outlier	N/A
[38]	✓	X	X	✓AE+	✓	outlier	N/A
[39]	✓	X	X	X	✓	outlier	99.43
[29]	✓	✓	✓	✓AE+	✓	outlier	97.66
[27]	✓	✓	X	✓AE	✓	outlier	N/A
[40]	✓	✓	X	X	✓	outlier	N/A
[30]	✓	✓	✓	AE	✓	outlier	N/A
[25]	✓	✓	X	AE	✓	novelty	N/A
[41]	X	✓	✓	X	✓	novelty	N/A
[42]	X	✓	X	X	X	novelty	98.59
[43]	✓	✓	X	X	X	novelty	N/A
[44]	✓	✓	X	X	X	novelty	N/A
[45]	✓	✓	X	X	X	novelty	99.00
[46]	✓	✓	X	X	✓	novelty	98.39
Proposed	✓	✓	✓	✓	✓	Outlier and novelty	99.72

The study articles' accuracy results were not disclosed in their published work, as shown by the “N/A″ in the “Accuracy (%)" column. When comparing the accuracy of other publications to the proposed research, the suggested study outperforms the bulk of the other articles in the table with an amazing accuracy rating of 99.72 %. While there are a few papers that achieved high accuracy percentages, such as 99.43 % [39] and 99.00 %[45], the proposed work still stands as one of the top performers in the realm of both novelty and outlier detection in time-series data, highlighting its strong capabilities and potential for real-world applications.

## 4 Conclusion

The DeepMaly technique introduced in this paper addresses the key difficulty of anomaly detection in Smart Home Systems (SHS) by offering a unique strategy for unsupervised anomaly identification inside time-series data. The approach identifies between seasonal and real abnormalities without the requirement for labeled datasets by combining Long Short-Term Memory (LSTM) and Deep Convolutional Neural Network (DCNN). The approach can deal with modest data contamination (less than 5 percent). Many other density and distance-dependent anomaly detection approaches often miss tiny deviations in the time-series dataset, but our methodology accurately detects them. DeepMaly was tested on three distinct datasets. The proposed method is validated with two public datasets: NAB realAdExchange and Yahoo A1. Considering the same benchmark dataset, the CNN network achieved comparatively higher results than the other LSTM and ML techniques. The proposed approach for detection can consider more variables in the proposed algorithm than the other existing techniques. It can be concluded from the evolutionary result that the proposed approach significantly impacts anomaly detection for time-series data so that specific information can be incorporated into the model with the time-series dataset. In addition, it has also shown that the unsupervised proposed model is also applicable to unlabeled real-time data. The suggested work is positioned as a strong challenger in novelty and outlier discovery within the time-series data environment, as indicated by its 99.72 % accuracy, significantly enhancing the toolkit for anomaly detection.

This method can be used in circumstances when there is a significant volume of data available but no way to identify it. On the other hand, poor data quality might sabotage the data modeling step. The system, however, will operate best if the pre-trained data has nearly no anomalous data, according to the novelty detection technique. Here, model expansion was focused on applying the domain adaptation notion to time series analysis anomaly detection. Working with deep hybrid learning for improved forecasting is also on the horizon, which would be beneficial.

## Data availability

Two time-series datasets were used in this study, which are NAB and Yahoo Webscope datasets. NAB dataset can be accessed using the link: https://doi.org/10.1109/ICMLA.2015.141. Yahoo Webscope dataset can be accessed using the link: https://webscope.sandbox.yahoo.com/catalog.php?datatype=s&did=70.

## CRediT authorship contribution statement

**Md Jakir Hossen:** Supervision, Data curation. **Jesmeen Mohd Zebaral Hoque:** Software, Methodology, Formal analysis, Conceptualization. **Nor Azlina binti Abdul Aziz:** Supervision, Funding acquisition. **Thirumalaimuthu Thirumalaiappan Ramanathan:** Validation, Resources, Investigation. **Joseph Emerson Raja:** Writing – review & editing.

## Declaration of competing interest

The authors declare the following financial interests/personal relationships which may be considered as potential competing interests:Dr. Md. Jakir Hossen reports financial support was provided by Universiti Multimedia. Md. Jakir Hossen reports a relationship with Universiti Multimedia that includes: employment.
